# Impact of movement tempo on bar velocity and time under tension in resistance exercises with different external loads

**DOI:** 10.5114/biolsport.2022.106160

**Published:** 2021-07-15

**Authors:** Robert Trybulski, Mariola Gepfert, Dawid Gawel, Marta Bichowska, Krzysztof Fostiak, Grzegorz Wojdala, Grzegorz Trybek, Michal Krzysztofik, Michal Wilk

**Affiliations:** 1 Department of Medical Sciences, The Wojciech Korfanty School of Economics, 40-065 Katowice, Poland; 2 Provita Zory Medical Center, 44-240 Zory, Poland; 3 Institute of Sport Sciences, Jerzy Kukuczka Academy of Physical Education in Katowice, Poland; 4 Faculty of Physical Education, Gdansk University of Physical Education and Sport, 80-336 Gdansk, Poland; 5 Department of Oral Surgery, Pomeranian Medical University, 72 Powstanców Wlkp. St., Szczecin, Poland

**Keywords:** Strength, Duration of Movement, Speed, Cadence, Contraction, Sport Performance

## Abstract

The goal of the study was to determine the differences between volitional and maximal movement tempo during resistance exercise. Ten healthy men volunteered for the study (age = 26.4 ± 4.8 years; body mass = 93.8 ± 9.6 kg; barbell squat one-repetition maximum (1RM) = 175 ± 16.7 kg; bench press 1RM = 140.5 ± 26.8 kg). In a randomized order, the participants performed six sets of the barbell squat and the bench press exercise at progressive loads from 40% to 90%1RM (step by 10%) under two testing conditions: with volitional movement tempo or with maximal movement tempo. The three-way repeated measures ANOVA showed a statistically significant multi-interaction effect for time under tension (p < 0.001), peak bar velocity (p = 0.04) and for mean bar velocity (p < 0.001). There was also a statistically significant main effect of movement tempo for time under tension (p < 0.001), peak bar velocity (p < 0.001) and for mean bar velocity (p < 0.001). The post hoc analysis for main effect of tempo revealed that time under tension was significantly longer for volitional compared to maximal tempo (0.84 vs 0.67 s, respectively), peak bar velocity was significantly higher for maximal compared to volitional tempo (1.24 m/s vs 0.90 m/s, respectively), and mean bar velocity was significant higher for maximal compared to volitional tempo (0.84 m/s vs 0.67 m/s, respectively). The presented results indicate that there were significant differences between volitional and maximal movement tempos in time under tension and bar velocity (peak and mean), as well as significant differences in those variables between the two exercises. Therefore, the velocity of movement and time under tension is related to movement tempo, external load and type of exercise used.

## INTRODUCTION

The tempo of movement of each repetition during resistance exercises is a training variable that has been extensively researched in recent years [1–4]. Movement tempo of particular repetitions is usually determined by the duration of one repetition (in s) or by the velocity of the movement (in m/s). The actual duration or velocity of movement (intentional or volitional), especially in the concentric phase depends on the external load used as well as on the appearance of fatigue symptoms [5, 6]. Increased load accompanied by fatigue extends the duration of movement tempo and decreases the velocity of movement [4, 7, 8] even when the movement tempo is intentional.

The changes in duration of the movement influence the number of performed repetitions [9, 10], time under tension [11], muscle activity [4, 12] and maximal load lifted during resistance exercise [13–15]. When the number of performed repetitions is the same, longer repetition duration results in greater total time under tension. The change in the duration of movement tempo influence training volume during a single training session, a training microcycle, or even a training mesocycle [15], what can also indirectly affect muscular strength and hypertrophy [1, 3, 16] due to changes in physiological responses such as hormone and blood lactate concentrations during and after resistance training [17–21]. However, acute responses following different movement tempos are also related to the level of experience in resistance training [22]. According to the American College of Sports Medicine [23] novice individuals should use slow and moderate movement tempos [24–29]. For intermediate individuals, it is recommended that moderate tempos should be used [24–29], while for advanced athletes a variety of tempos from slow to fast velocities are recommended. However, these recommendations do not specify which values of movement duration are slow, moderate or fast. Furthermore there is no data about the duration of volitional movement tempo against different loads. Jandačka and Beremlijski [30], as well as Izquierdo et al. [5] showed that a relationship exists between bar velocity, number of preformed repetitions and the external load used. However, these analysis were performed only with the use of maximal movement tempo and currently there is no scientific data confirming such relationships during volitional movement tempo.

Despite that, the movement tempo is an important variable in resistance training many scientific studies still do not determine and do not register this variable and use volitional movement tempo (not controlled) during research procedures without precisely determining its value [31, 32]. Thus, not specifying the duration of movement tempo may be a significant limitation in resistance exercise studies, and may be the cause of conflicting results in terms of the effectiveness of resistance training directed at the development of hypertrophy, strength or power [33]. Therefore, the aim of this study was to determine the duration and velocity of movement that occurs during volitional movement tempo. A second aim of the study was to determine the difference between volitional and maximum movement tempo and to determine the relationship between these changes in relation to the type of exercise and external load used. Since the bench press and squats are commonly used exercises in experimental research and practice, and are also recognized as effective in the development of the upper and lower body [34, 35], these exercises were selected for this study.

## MATERIALS AND METHODS

In this study a randomized crossover design experiment has been used, the participants performed two training sessions consisting of a squat and bench press exercises with 6 sets of 1 repetition in each exercise, with volitional (V/0/V/0) or maximal movement tempo (X/0/X/0). To determine the duration and velocity of movement, squat and bench press exercises and loads from 40% to 90%1RM (steps by 10%) were used. During the experimental procedures 5-minute rest intervals between sets were used, and a 10-minute rest interval between exercises. All testing sessions were performed in the Strength and Power Laboratory at the Academy of Physical Education in Katowice, Poland as well as in University of Physical Education and Sport in Gdansk. The study protocol was approved by the Bioethics Committee for Scientific Research, at the Academy of Physical Education in Katowice, Poland (10/2018), in accordance with the ethical standards of the Declaration of Helsinki, 1983.

### Participants

Ten healthy, resistance trained men volunteered for the study (age = 26.4 ± 4.8 years; body mass = 93.8 ± 9.6 kg; experience in resistance training 7.7 ± 4.3 years; barbell squat one-repetition maximum (1RM) = 175 ± 16.7 kg; bench press 1RM = 140.5 ± 26.8 kg). The following inclusion criteria were used: a) 1RM squat of at least 150% own body mass, and 1RM bench press of at least 120% own body mass, b) no musculoskeletal injuries prior to the examination, c) experience in resistance training with different movement tempos. Participants were informed about the benefits and potential risks of the study and gave written informed consent to participate in the study.

### Procedures

#### Familiarization Session

Two weeks before the main experiment, the participants performed a familiarization session. First, the participants performed a general body warm-up, followed by a specific resistance exercise warm-up performed at a load of 20 and 40% of their estimated 1RM. After the warm-up, the familiarization session began. During the familiarization session, each participant performed 4 sets of 1 repetition of the barbell squat and bench press exercises at a load from 50–80% of their estimated 1RM with a 10% load progression. Two of those sets were performed with volitional and 2 sets with maximal movement tempo.

#### 1RM Strength Test

One week before the main experiment the 1RM squat and bench press test was performed as described elsewhere [14, 35]. During the 1RM test session, the general body warm-up was the same as during the familiarization session. Afterward, the participants first performed specific squat warm-up repetitions at a load of 20, 40, and 60% of their estimated 1RM. The first testing load was set to an estimated 80%1RM and was increased by 2.5 to 10 kg for each subsequent trial. This process was repeated until failure. After completing the 1RM test procedure for the squat exercise, and a 10 minutes rest interval, the 1RM bench press test began. The 1RM testing procedure for bench press was the same as for the squat exercise. During the 1RM test, the participants executed one repetition with a volitional movement tempo [14, 15]. The rest interval between successful sets was 5 min. Grip width on the bar was set at 150% of the individual bi-acromial distance, and this was used for all main attempts and all experimental sessions [11].

#### Experimental Sessions

In a randomized and counterbalanced order, the participants performed the barbell squat and bench press exercises. During experimental sessions the participants performed six sets of each exercise at loads from 40% to 90%1RM (load progression by 10%) under two testing conditions: with volitional movement tempo or with maximal movement tempo in both, the eccentric and concentric phases of movement. A Tendo Power Analyzer system (Tendo Sport Machines, Trencin, Slovakia) was used for the evaluation of concentric time under tension and concentric bar velocity. Measurements were made independently for each repetition and automatically converted into values of time under tension (s) peak bar velocity (m/s), and mean bar velocity (m/s).

### Statistical Analysis

All statistical analysis were performed using Statistica 9.1. Results are presented as means with standard deviations. The Shapiro-Wilk, test was used in order to verify the normality, homogeneity and sphericity of the sample data variances, respectively. To evaluate differences in measurements between volitional and maximal movement tempo for squats and the bench press exercise, we used a three-way ANOVA (2 exercises × 2 tempos × 6 sets). The statistical significance was set at *p* < 0.05. The effect size was determined by partial eta squared (η^2^). Partial eta squared values were classified as small (0.01 to 0.059), moderate (0.06 to 0.137) and large (> 0.137) [36]. The Tukey’s test was conducted to determine the differences between mean values. Parametric effect sizes were defined as: large (g > 0.8); moderate (g between 0.8 and 0.5); small (g between 0.49 and 0.20) and trivial (g < 0.2) [37]. Percent changes with 95% confidence intervals (95CI) were also calculated.

## RESULTS

The three-way repeated measures ANOVA showed a statistically significant multi-interaction effect for time under tension (p < 0.001; η2 = 0.35), peak bar velocity (p = 0.04; η2 = 0.24) and for mean bar velocity (p < 0.001; η2 = 0.34). The post hoc analysis for multi-interaction are presented in table 1.

**TABLE 1:** Comparisons between the experimental conditions and particular loads for all measured variables.

	40%1RM (95%CI)	50%1RM (95%CI)	60%1RM (95%CI)	70%1RM (95%CI)	80%1RM (95%CI)	90%1RM (95%CI)
**Condition**	**Time Under Tension concentric (s)**					
Barbell Squat	0.61 ± 0.07	0.67 ± 0.08	0.73 ± 0.06	0.83 ± 0.10	0.95 ± 0.15	1.11 ± 0.18
Volitional tempo	(0.56 to 0.66)	(0.61 to 0.73)	(0.69 to 0.78)	(0.76 to 0.90)	(0.84 to 1.05) ^#^	(0.98 to 1.24) ^#^
Barbell Squat	0.49 ± 0.07	0.54 ± 0.07	0.61 ± 0.09	0.68 ± 0.10	0.80 ± 0.15	0.98 ± 0.16
Maximal tempo	(0.44 to 0.54)	(0.49 to 0.59)	(0.54 to 0.67)	(0.61 to 0.75)	(0.69 to 0.91)	(0.86 to 1.10)
Mean differences	0.12	0.13	0.12	0.15	0.15	0.13
Effect Size	1.71	1.73	1.57	1.50	1.00	0.76
Bench Press	0.47 ± 0.05	0.55 ± 0.05	0.68 ± 0.12	0.87 ± 0.25	1.13 ± 0.32	1.43 ± 0.31
Volitional tempo	(0.43 to 0.51)	(0.51 to 0.58)	(0.59 to 0.76)	(0.69 to 1.05) *	(0.90 to 1.36) *^#^	(1.21 to 1.65)* ^#^
Bench Press	0.41 ± 0.02	0.49 ± 0.03	0.58 ± 0.06	0.64 ± 0.07	0.83 ± 0.05	1.01 ± 0.42
Maximal Tempo	(0.39 to 0.42)	(0.47 to 0.51)	(0.53 to 0.63)	(0.60 to 0.69) *	(0.80 to 0.87) *	(0.71 to 1.31) *
Mean differences	0.06	0.06	0.10	0.23*	0.30*	0.42*
Effect Size	1.58	1.46	1.05	1.25	1.31	1.14

**Mean Bar Velocity (m/s)**						
Barbell Squat	0.76 ± 0.11	0.69 ± 0.10	0.64 ± 0.08	0.58 ± 0.07	0.51 ± 0.07	0.44 ± 0.06
Volitional tempo	(0.68 to 0.84) *	(0.62 to 0.76) *^#^	(0.58 to 0.70)* ^#^	(0.53 to 0.63) *^#^	(0.46 to 0.55)*^#^	(0.39 to 0.48)* ^#^
Barbell Squat	1.05 ± 0.10	0.96 ± 0.11	0.85 ± 0.09	0.75 ± 0.07	0.66 ± 0.07	0.54 ± 0.06
Maximal tempo	(0.98 to 1.13) *	(0.89 to 1.04) *	(0.78 to 0.92)*	(0.70 to 0.80)* ^	(0.61 to 0.71)* ^	(0.49 to 0.58) *^
Mean differences	0.29*	0.27*	0.21*	0.17*	0.15*	0.10*
Effect Size	2.76	2.57	2.47	2.43	2.14	1.67
Bench Press	0.70 ± 0.08	0.61 ± 0.05	0.53 ± 0.05	0.42 ± 0.07	0.35 ± 0.05	0.24 ± 0.04
Volitional tempo	(0.65 to 0.76)*	(0.57 to 0.64) *^#^	(0.49 to 0.56)* ^#^	(0.37 to 0.48)* ^#^	(0.31 to 0.39) *^#^	(0.21 to 0.26) ^#^
Bench Press	1.11 ± 0.11	0.96 ± 0.09	0.79 ± 0.13	0.65 ± 0.11	0.44 ± 0.10	0.29 ± 0.06
Maximal Tempo	(1.03 to 1.18) *	(0.89 to 1.03) *	(0.70 to 0.87) *	(0.58 to 0.73)* ^	(0.37 to 0.51)* ^	(0.25 to 0.33)^
Mean differences	0.41*	0.35*	0.26*	0.23*	0.09*	0.05
Effect Size	4.26	4.81	2.65	2.49	1.14	0.98

**Peak Bar Velocity (m/s)**						
Barbell Squat	1.26 ± 0.21	1.19 ± 0.23	1.15 ± 0.23	1.11 ± 0.15	1.03 ± 0.21	1.02 ± 0.23
Volitional tempo	(1.11 to 1.41)* ^#^	(1.02 to 1.35)* ^#^	(0.99 to 1.32)* ^#^	(0.99 to 1.22)*^#^	(0.88 to 1.18)*^#^	(0.85 to 1.18)*^#^
Barbell Squat	1.75 ± 0.22	1.69 ± 0.23	1.52 ± 0.16	1.43 ± 0.14	1.32 ± 0.11	1.20 ± 0.12
Maximal tempo	(1.60 to 1.91)*^	(1.53 to 1.86)*^	(1.41 to 1.63)*^	(1.33 to 1.52)*^	(1.24 to 1.40)*^	(1.12 to 1.28)*^
Mean differences	0.49*	0.50*	0.37*	0.32*	0.29*	0.18*
Effect Size	2.28	2.17	1.87	2.21	1.73	0.98
Bench Press	1.06 ± 0.15	0.86 ± 0.09	0.71 ± 0.08	0.59 ± 0.09	0.48 ± 0.08	0.38 ± 0.06
Volitional tempo	(0.95 to 1.17)* ^#^	(0.80 to 0.92)* ^#^	(0.65 to 0.77) ^#^*	(0.52 to 0.66)* ^#^	(0.43 to 0.54) ^#^	(0.33 to 0.42)^#^
Bench Press	1.59 ± 0.21	1.41 ± 0.22	1.08 ± 0.26	0.90 ± 0.19	0.61 ± 0.15	0.45 ± 0.09
Maximal Tempo	(1.44 to 1.74)*^	(1.25 to 1.56)*^	(0.90 to 1.27)*^	(0.76 to 1.03)*^	(0.50 to 0.71)^	(0.38 to 0.51)^
Mean differences	0.53*	0.55*	0.37*	0.31*	0.13	0.07
Effect Size	2.9	3.27	1.92	2.09	1.08	0.92

Results are mean ± SD (95% confidence intervals).

* Significant differences between volitional and maximal movement tempo in exercise;

# significant differences between exercise in volitional movement tempo

^ significant differences between exercise in maximal movement tempo.

The three-way repeated measures ANOVA showed a statistically significant main effect of movement tempo (volitional vs maximal) for time under tension (p < 0.001; η2 = 0.90), peak bar velocity (p < 0.001; η2 = 0.88) and for mean bar velocity (p < 0.001; η2 = 0.94). There was also significant main effect of exercise (squat vs bench press) for peak bar velocity (p < 0.001; η2 = 0.91) and mean bar velocity (p < 0.001; η2 = 0.73) but not for time under tension (p = 0.85; η2 = 0.004). The post hoc analysis for main effect of tempo revealed that time under tension was significantly longer for volitional compared to maximum movement tempo (p < 0.001; 0.84 vs 0.67 s, respectively), peak bar velocity was significantly higher for maximal compared to volitional movement tempo (p < 0.001; 1.24 m/s vs 0.90 m/s, respectively), and mean bar velocity was significant higher for maximal compared to volitional tempo (p < 0.001; 0.84 m/s vs 0.67 m/s, respectively). The post hoc analysis for main effect of exercise revealed that peak bar velocity was significantly higher for the squat compared to the bench press exercise (p < 0.001; 1.31 m/s vs 0.84 m/s, respectively), and mean bar velocity was significantly higher for the squat compared to the bench press exercise (p < 0.001; 0.75 m/s vs 0.59 m/s, respectively).

## DISCUSSION

The main findings of the study indicated that they were significant differences between volitional and maximal movement tempo in time under tension and bar velocity (peak and mean). Regardless of the volitional or maximal movement tempo, increases load caused a decrease in bar velocity. However, with increased external load, the differences in bar velocity between volitional and maximal movement tempos decreased. Furthermore, we observed a significantly higher bar velocity across all loads during the barbell squat, as compared to the bench press exercise. Therefore, the velocity of movement and differences between movement tempos were related to external loads and the type of exercise used. Additionally, the increased load caused an extension of time under tension for both maximal and volitional movement tempo. However, the differences in time under tension between volitional and maximal movement tempo in the squat exercise were constant for all external loads used (MD = 0.12 to 0.15 s), while during the bench press exercise the time under tension increased with progressive loads (MD = 0.06 to 0.42 s). Thus similar as to bar velocity, the time under tension and differences between volitional and maximal movement tempos were related to external load and to the type of resistance exercise used.

The result of the presented research showed that the velocity of movement during the volitional tempo was within 0.88 m/s and 0.30 to 1.55 m/s for mean and peak values respectively, and with-in 0.41 to 2.14 s for time under tension (table 2). However, these variables differed significantly compared to the maximum tempo of movement in which the velocity ranged from 0.22 to 1.31 m/s and from 0.35 to 2.10 m/s for mean and peak values, respectively, and in the range of 0.38 to 1.44 s for time under tension (Table 2). Furthermore, the bar velocity and time under tension varied depending on the external load as well as the type of exercise used. Jandačka and Beremlijski [30] indicated a significant relationship between bar velocity and external load used as well as a relationship between bar velocity and type of exercise used. A similar relationship was demonstrated between maximal velocity of movement and the degree of muscle failure [5]. However, those analysis were performed only with maximal movement tempo, and currently there is no scientific data on whether such relationships also occur during volitional movement tempo. The results of the presented research are the first to indicate that bar velocity decreases with increasing external load not only during maximal movement tempo but also when exercises are performed at volitional movement tempo. Therefore, the results of this study are in agreement with previous studies, where we registered a natural decrease of velocity in successive repetitions in a particular set [38, 39]. Additionally, the results of this study showed that the time under tension in single repetition also depend on the external load used. When the external load increases the time under tension also increases, in both maximal as well as volitional tempo of movement. Therefore, not only is there a velocity and external load time-course relationship, but also a time under tension and load time-course dependence. Such time-course dependencies were observed in the squat and bench press exercises.

**TABLE 2:** The minimum – maximum range for all measured variables.

	40%1RM (min; max)	50%1RM (min; max)	60%1RM (min; max)	70%1RM (min; max)	80%1RM (min; max)	90%1RM (min; max)
**Condition**	**Time Under Tension concentric (s)**					
Barbell Squat	0.51	0.54	0.65	0.75	0.76	0.91
Volitional tempo	0.71	0.75	0.83	1.05	1.29	1.50
Barbell Squat	0.41	0.45	0.44	0.54	0.57	0.69
Maximal tempo	0.64	0.70	0.77	0.83	1.00	1.22
Bench Press	0.41	0.49	0.56	0.64	0.90	1.15
Volitional tempo	0.55	0.68	0.99	1.55	1.93	2.14
Bench Press	0.38	0.45	0.50	0.57	0.77	0.10
Maximal Tempo	0.46	0.54	0.72	0.79	0.94	1.44

**Mean Bar Velocity (m/s)**						
Barbell Squat	0.56	0.48	0.47	0.48	0.40	0.36
Volitional tempo	0.88	0.81	0.78	0.70	0.60	0.54
Barbell Squat	0.90	0.77	0.70	0.61	0.55	0.43
Maximal tempo	1.26	1.13	0.99	0.86	0.76	0.62
Bench Press	0.57	0.50	0.45	0.27	0.21	0.17
Volitional tempo	0.85	0.68	0.59	0.49	0.40	0.29
Bench Press	0.98	0.85	0.58	0.50	0.33	0.22
Maximal Tempo	1.31	1.11	0.97	0.79	0.61	0.40

**Peak Bar Velocity (m/s)**						
Barbell Squat	0.85	0.70	0.70	0.93	0.63	0.72
Volitional tempo	1.55	1.41	1.51	1.42	1.30	1.46
Barbell Squat	1.42	1.26	1.21	1.13	1.11	0.99
Maximal tempo	2.10	2.08	1.68	1.56	1.48	1.38
Bench Press	0.82	0.74	0.56	0.42	0.36	0.30
Volitional tempo	1.36	1.01	0.82	0.71	0.59	0.50
Bench Press	1.38	1.10	0.64	0.62	0.45	0.35
Maximal Tempo	1.96	1.68	1.44	1.17	0.87	0.61

Furthermore, the results of the presented research showed significant differences in bar velocity between exercises, independently for volitional and maximal movement tempo. When exercise was performed with volitional movement tempo, peak bar velocity was significantly higher for the squat compared to bench press exercise at all load (40–90%1RM). Similarly, mean bar velocity under volitional movement tempo was significantly higher for the squat compared to the bench press exercise at all loads except for 40%1RM. When exercise was performed with maximal movement tempo peak bar velocity was also higher for the squat compared to the bench press exercise for all loads, however for mean bar velocity such differences were observed only at higher loads (70–90%1RM). Therefore, a different pattern of velocity declines was observed between the two exercises tested. Previous studies showed that the time-course decreases in unintentional movement velocity, and may vary between the upper and lower extremity [30]. Such differences may be related with biomechanical characteristics of the kinetic chain of the exercises or to different muscle fiber distribution between particular muscle groups [5]. Therefore, the time-course of bar velocity observed in the presented study was related with the type of exercise used, what is consistent with previous research [30].

Furthermore, when exercises were performed with maximal movement tempo the relative velocity decreased between 40% and 90%1RM, and occurred at a greater rate in the bench press than in the squat exercise (table 3) what is consistent with the study of Izquierdo et al. [5]. Similarly, relative velocity decreased when the exercises were performed with volitional movement tempo, and a higher decrease of bar velocity was observed for the bench press compared to the squat exercise. On the contrary the time under tension for loads 40 to 90%1RM increases at a greater rate in the bench press than in the squat exercise. What is particularly important, relative changes between 40 and 90%1RM were lower for the volitional compared to the maximal movement tempo. Therefore, the velocity and time under tension during the resistance exercise are related with external load used, type of exercise but also with the tempo of movement used. The possible explanation for the different pattern of decline in time under tension and bar velocity between the squat and bench press exercise may be associated with extremity-related differences in maximal strength, muscle cross-sectional area, fiber type distribution [40], muscle mechanics (i.e. length and muscle pennation angle) of the upper and lower limbs, together with functional differences according to the joint position and geometry of the joints and levers [5, 39].

**TABLE 3:** The differences between 40% and 90%1RM for all measured variables.

	40%1RM	90%1RM	Mean differences	Relative differences (%)
**Time Under Tension (s)**				
Barbell Squat Volitional tempo	0.61 ± 0.07*	1.11 ± 0.18*	0.50	81.97
Barbell Squat Maximal tempo	0.49 ± 0.07*	0.98 ± 0.16*	0.49	100.00
Bench Press Volitional tempo	0.47 ± 0.05*	1.43 ± 0.31*	0.96	204.26
Bench Press Maximal Tempo	0.41 ± 0.02*	1.01 ± 0.42*	0.60	146.34

**Mean Bar Velocity (m/s)**				
Barbell Squat Volitional tempo	0.76 ± 0.11*	0.44 ± 0.06*	0.32	-42.11
Barbell Squat Maximal tempo	1.05 ± 0.10*	0.54 ± 0.06*	0.51	-48.57
Bench Press Volitional tempo	0.70 ± 0.08*	0.24 ± 0.04*	0.46	-65.71
Bench Press Maximal Tempo	1.11 ± 0.11*	0.29 ± 0.06*	0.82	-73.87

**Peak Bar Velocity (m/s)**				
Barbell Squat Volitional tempo	1.26 ± 0.21*	1.02 ± 0.23*	0.24	-19.05
Barbell Squat Maximal tempo	1.75 ± 0.22*	1.20 ± 0.12*	0.55	-31.43
Bench Press Volitional tempo	1.06 ± 0.15*	0.38 ± 0.06*	0.68	-64.15
Bench Press Maximal Tempo	1.59 ± 0.21*	0.45 ± 0.09*	1.14	-71.70

* Significant differences between 40% and 90%1RM.

Despite the uniqueness of the presented results, there were several limitations in the experimental design employed which should be addressed to understand the significance of the outcomes. The level of experience in resistance exercise has a significant impact on maximal as well volitional bar velocity and time under tension [22]. Therefore, the results of this study only apply to participants who are advanced in resistance training, and may not translate into beginners in resistance exercise. Moreover, the research procedure did not include the analysis of variables during the eccentric phase of movement. According to Wilk et al. [14, 41] the tempo of movement during eccentric contraction has a significant impact on bar velocity during the concentric contraction.

## CONCLUSIONS

The results of the present study indicate that were significant differences between volitional and maximal movement tempo in time under tension and bar velocity. Furthermore, the increases of external load caused decreases in bar velocity. However, there was a different pattern of velocity changes between movement tempo as well as between the exercises tested. Therefore. the velocity of movement, time under tension for maximal as well as for volitional movement tempo were related to the external load and the type of exercise used.
